# Evaluation of Reaction Time and Hand–Eye Coordination in Schoolchildren Using Wearable Sensor-Based Systems: A Study with Neural Trainer Devices

**DOI:** 10.3390/s25227006

**Published:** 2025-11-17

**Authors:** José Alfredo Sulla-Torres, Nadia Yunorvi Chavez-Salas, María Fernanda Valverde-Riveros, Diego Alonso Iquira-Becerra, Karina Rosas-Paredes, Marco Antonio Cossio-Bolaños

**Affiliations:** 1 Escuela Profesional de Ingeniería de Sistemas, Universidad Católica de Santa María, Arequipa 04001, Peru; 2 Programa de Doctorado en Ciencias de la Actividad Física, Universidad Católica del Maule, Talca 3466706, Chile

**Keywords:** reaction time, hand–eye coordination, wearable technology, children, sensors, neural trainer

## Abstract

Reaction time and hand–eye coordination are critical neuromotor skills in school-aged children, influencing academic, cognitive, and motor development. The objective of this study was to evaluate schoolchildren’s performance on reaction time tests using Neural Trainer device sensors and wearable technology, establishing baseline metrics and identifying lateral performance asymmetries. Fifty-nine schoolchildren performed six sensor-based motor tests involving bimanual and unimanual interaction: P1 (10 timed repetitions, bimanual), P2 (10 timed repetitions, left hand), P3 (10 timed repetitions, right hand), P4 (hits, bimanual), P5 (hits, left hand), and P6 (hits, right hand). Neural Trainer devices with four light nodes were used for activity monitoring. Data was analyzed using statistical methods to assess time, accuracy, and variability. The results showed that the average times were P1 = 8.69 ± 1.44 s, P2 = 8.90 ± 1.30 s, and P3 = 8.83 ± 1.29 s. The average successes were P4 = 22.90 ± 3.10, P5 = 22.00 ± 3.40, and P6 = 24.42 ± 2.72 hits. Significant differences were found between hands in successes (*p* < 0.001) but not in times (*p* = 0.716). The ANOVA for the hit trials revealed significant differences between conditions, F(2, 174) = 9.30, *p* < 0.001. The conclusions indicate that sensor-based systems such as the Neural Trainer device demonstrated the potential to provide objective and consistent measurements of reaction time in schoolchildren; however, further studies comparing its performance with established clinical assessment tools are necessary to confirm its validity and diagnostic accuracy.

## 1. Introduction

Reaction time and hand–eye coordination are fundamental components of neuromotor development in school-aged children. These skills have a direct impact on academic performance, fine motor skill development, and functional activities of daily living [1]. Objective assessment of these abilities requires precise instruments and standardized methodologies that enable reliable, reproducible measurements [2].

Brito et al. [3] reported that neurofeedback and biofeedback programs, complemented by wearable technology, accelerate decision-making, response speed, and visuomotor accuracy in young populations. Studies such as [4] designed measuring tools for hand–eye coordination based on sensor technology. The subject performs a movement with both hands open to turn off or press the activated sensor randomly. The test takes 60 s; the score is the best of two repetitions. In [5], they explored the use of visual stimuli to measure rapid upper-limb movements, demonstrating that perception–action devices can effectively predict reaction times (RTs) and thus performance in field agility tests. Rasmussen et al. [6] evaluated the Motus wearable sensor-based system for accurately classifying postures and movements in children aged 3–14 years, demonstrating the feasibility of personalized, real-time monitoring.

In recent decades, advances in wearable technologies and smart sensors have radically transformed the assessment of neuromotor function. Devices such as the Neural Trainer, equipped with tactile and motion sensors, have proven to be practical tools for capturing millisecond temporal data, quantifying errors, and recording motor responses with high accuracy [7] and wearable technology [8]. These systems offer significant advantages over traditional methods, including increased measurement accuracy, real-time recording, and the ability to perform multidimensional performance analysis [9]. These studies, with preliminary results, demonstrated that these devices can be used for accurate psychophysical measurements and that their systematic use improves key perceptual–motor aspects of academic and athletic development in school-age children [10].

Hand–eye coordination is a critical area of child development, closely linked to skills such as writing, tool use, and manipulation of school materials. Alterations in this skill may be associated with learning difficulties, developmental disorders, or specific neurological conditions [11]. In this sense, the implementation of advanced technological measurement systems, capable of identifying subtle variations in motor coordination, enables not only earlier diagnoses but also the design of more effective educational and clinical interventions [12]. Recent advances in neurotechnological interfaces and wearable systems have further expanded the possibilities for multimodal neuromotor assessment, enabling the integration of biosignals, motion tracking, and cognitive data into unified analysis frameworks [13].

The present study aims to evaluate schoolchildren’s performance on specific tests of reaction time and hand–eye coordination using Neural Trainer devices and low-latency wearable sensors. Differences in performance will be analyzed according to execution modality (bimanual vs. unimanual) and laterality (left hand vs. right hand), and normative parameters will be established that may be useful for future clinical, educational, or sports applications.

## 2. Materials and Methods

### 2.1. Study Design

A cross-sectional, quantitative, experimental study was conducted. The design enabled the systematic assessment of reaction time in schoolchildren under controlled, standardized conditions.

### 2.2. Participants

The sample consisted of 59 schoolchildren (N = 59) selected through non-probability convenience sampling. The inclusion criteria were school age (6–12 years), absence of diagnosed motor or cognitive difficulties, and informed consent from parents or guardians, in accordance with the Declaration of Helsinki. Participants with uncorrected visual impairments or upper limb injuries were excluded.

### 2.3. Instrumentation

Neural Trainer [14] devices comprise a mobile app (v. 1.2.19 for Android) that communicates with various external modules, referred to as “nodes.” Each node features movement sensors and an LED-based screen front that can be activated with a variety of visual stimulus patterns, including numbers, letters, different colors, and shapes. Each node has an accelerometer, gyroscope, and magnetometer, which measure the body’s linear acceleration, angular rotation, and spatial orientation. This data allows the user’s reaction time, response times, coordination, and movements to be calculated during an exercise or test.

In this way, the system enables the planning and development of training sessions that combine cerebral and motor stimuli, thereby enhancing the athlete’s performance and facilitating the development of new skills, such as rapid decision-making.

The standard configuration of the Neural Trainer device included

- Visual activity with red color selection only;
- Four nodes per station, approximately 20 cm apart;
- Motion sensor set to low mode;
- Light duration: 10 s;
- Start with an automatic countdown.

### 2.4. Evaluation Protocol

#### 2.4.1. Evaluation Conditions

Participants stood in front of the lights placed at belly level, keeping their hands in a predetermined position on the table. Subjects were instructed to move their hand over the light that would turn on the red light and return to the starting position as quickly as possible.

#### 2.4.2. Implemented Tests

(a) First evaluation: Time to complete 10 repetitions

**Figure 1:**
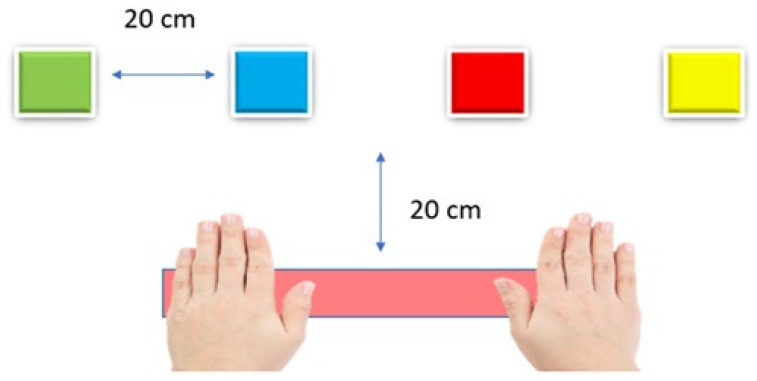
Neural Trainer Node layout for the test.

The protocol used for the reaction time test was as follows:

The subject was asked to stand in front of the lights, placed at belly level. Each light was approximately 20 cm apart. Their hands were placed on the table at the level of the lights, each with a predetermined mark. Hits refer to the number of correct sensor activations performed within the 15 s test period. Figure 1 shows the representation:

The subject is asked to move one hand over the spotlight that turns on a red light and return to the starting position as quickly as possible, repeating the movement for 10 repetitions without stopping. The test will be performed with the left hand only, the right hand only, and both hands.

The time taken to complete the 10 repetitions, the number of hits (correct answers), and the average reaction time will be recorded.

(b) Second evaluation: Number of hits in 15 s

The subject is asked to stand in front of the lights, which will be placed at belly level. Each light is approximately 20 cm apart. The hands will be placed on the table at the level of the lights with a predetermined mark.

The arrangement of the neural nodes and hands is as shown in Figure 1.

The subject is asked to move one hand over the light that turns on the red light and return to the starting position as quickly as possible, repeating the movement for 15 s without stopping. The test will be performed with only the left hand, only the right hand, and with both hands.

The hits, errors, and average reaction time will be recorded during the 15 s test.

The collected variables are shown below:

Test P1—10 timed repetitions, bimanual
  - Duration: 10 repetitions;
  - Modality: Both hands;
  - Variable measured: Total time in seconds;
  - Record: Time, hits, and average reaction time.

Test P2—10 timed repetitions, left hand
  - Duration: 10 repetitions;
  - Modality: Left hand only;
  - Variable measured: Total time in seconds;
  - Record: Time, hits, and average reaction time.

**Figure 2:**
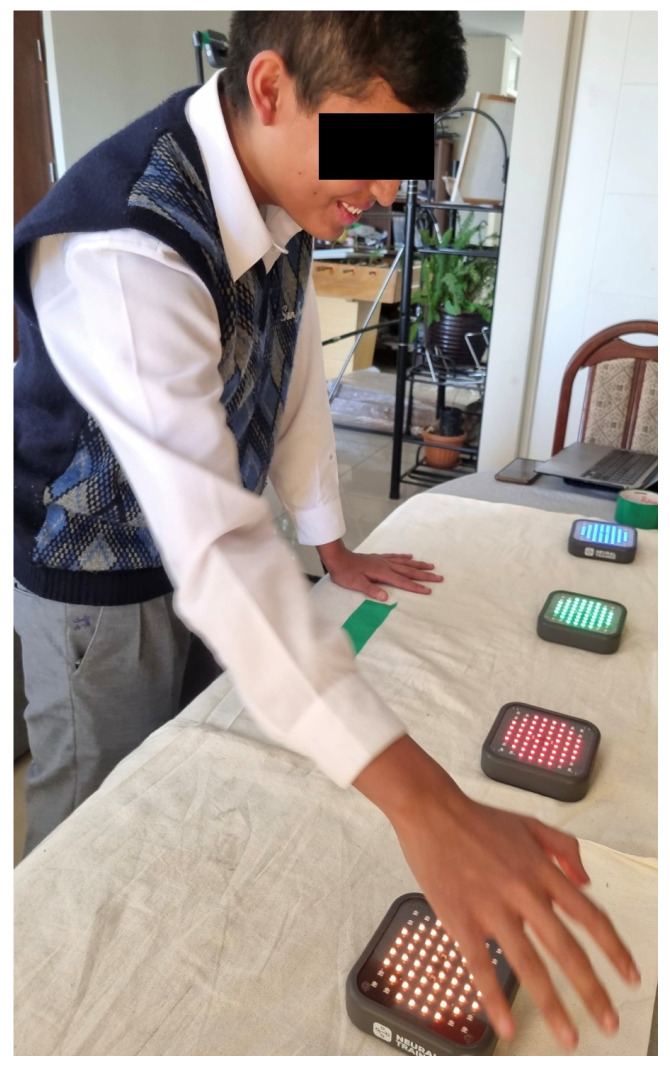
Test P3—10 timed repetitions, right hand.

Test P3—10 timed repetitions, right hand (see Figure 2)
  - Duration: 10 repetitions;
  - Modality: Right hand only;
  - Variable measured: Total time in seconds;
  - Record: Time, hits, and average reaction time.

Test P4—Hits, bimanual
  - Duration: 15 s;
  - Modality: Both hands;
  - Variable measured: Number of hits;
  - Record: Hits, errors, and average reaction time.

Test P5—Hits, left hand
  - Duration: 15 s;
  - Modality: Left hand only;
  - Variable measured: Number of hits;
  - Record: Hits, errors, and average reaction time.

Test P6—Hits, right hand
  - Duration: 15 s;
  - Modality: Right hand only;
  - Variable measured: Number of hits;
  - Record: Hits, errors, and average reaction time.

### 2.5. Statistical Analysis

The data were processed using Python 3.10, along with the pandas, numpy, and scipy libraries.stats, matplotlib, and seaborn libraries. Descriptive analyses (mean, standard deviation, percentiles), normality tests (Shapiro–Wilk), Pearson correlation analysis, Student’s *t*-tests for related samples, and one-way ANOVA were performed. A *p*-value < 0.05 was considered significant.

## 3. Results

### 3.1. Descriptive Statistics

Table 1 presents the complete descriptive statistics for all tests performed. The data show a relatively homogeneous distribution of reaction times, with greater variability in the hits. These values represent the average number of hits (±SD) per test (P4–P6), complementing the analysis of reaction time and providing a more complete representation of participants’ performance.

### 3.2. Data Distribution Analysis

Shapiro–Wilk normality tests revealed that most variables did not follow a normal distribution (*p* < 0.05), except for P6 (W = 0.963, *p* = 0.070). This information was taken into consideration when selecting the appropriate statistical tests.

### 3.3. Correlation Analysis

**Figure 3:**
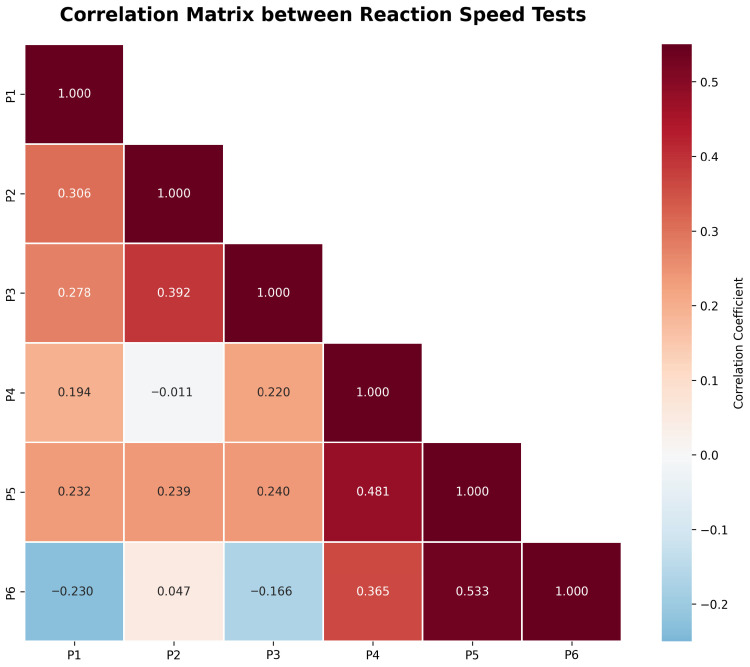
Correlation matrix between reaction time tests.

The correlation matrix (Figure 3) showed moderate correlations among the variables. The most notable correlations were as follows:

- P5 (15 s hits, left hand) and P6 (15 s hits, right hand) (r = 0.533, *p* < 0.001): moderate positive correlation between left- and right-handed guesses.
- P4 (15 s hits, bimanual) and P5 (15 s hits, left hand) (r = 0.481, *p* < 0.001): moderate positive correlation between bimanual and left-handed guesses.
- P2 (10-repetition time, left hand) and P3 (10-repetition time, right hand) (r = 0.392, *p* < 0.01): moderate positive correlation between left- and right-handed guesses.

The interpretation of correlation strength follows Cohen’s conventional thresholds [15]: r < 0.30 = weak, 0.30 ≤ r < 0.50 = moderate, and r ≥ 0.50 = strong. Accordingly, the correlations reported in Section 3.3 (r = 0.392–0.533) fall within the moderate range under this criterion.

The positive correlations indicate that participants who performed better (i.e., faster times or higher hits) with one hand tended to show similar performance with the other hand or under bimanual conditions. This suggests the presence of shared neuromotor coordination mechanisms and symmetrical development of reaction abilities. Conversely, a negative correlation—if observed—would indicate a compensatory or asymmetric performance pattern between limbs.

### 3.4. Analysis of Lateral Differences

#### 3.4.1. Comparison of Times (P2 vs. P3)

The paired-sample *t*-test revealed no significant difference in execution time between the left and right hands (t = 0.366, *p* = 0.716). This indicates that both hands exhibited similar temporal performances in the 10-repetition tests.

**Figure 4:**
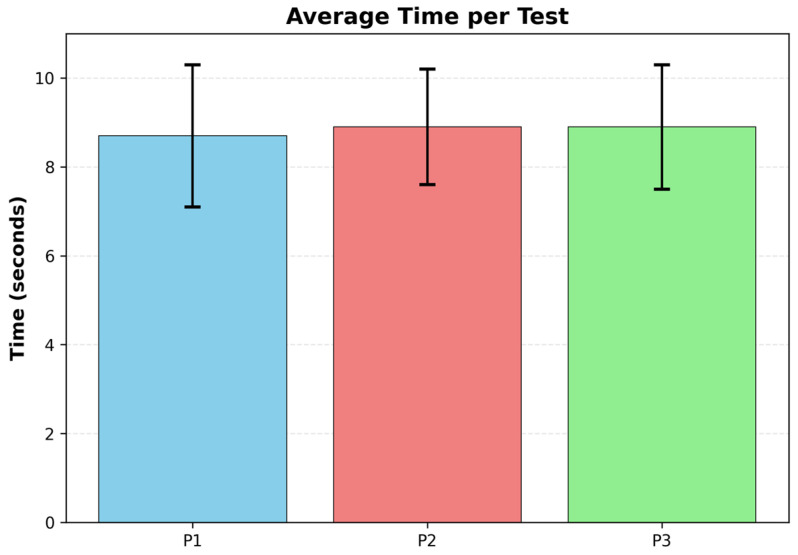
Results of the comparison of the average time per test.

Figure 4 shows the average time comparison for each test.

#### 3.4.2. Comparison of Hits (P5 vs. P6)

Highly significant differences were found between the hits of the left and right hands using a paired-sample *t*-test (t(87) = −6.168, *p* < 0.001, Cohen’s d = 0.657). The right hand showed superior performance (M = 24.42, SD = 2.72) compared to the left hand (M = 22.00, SD = 3.40), with an average difference of 2.42 hits. The observed right-hand superiority in accuracy (P5–P6) should be interpreted cautiously, as it may reflect the predominance of right-handed participants in the sample rather than an inherent neuromotor asymmetry. Stratification by hand, age, and prior motor experience is recommended in future studies to better isolate the sources of lateral performance differences.

**Figure 5:**
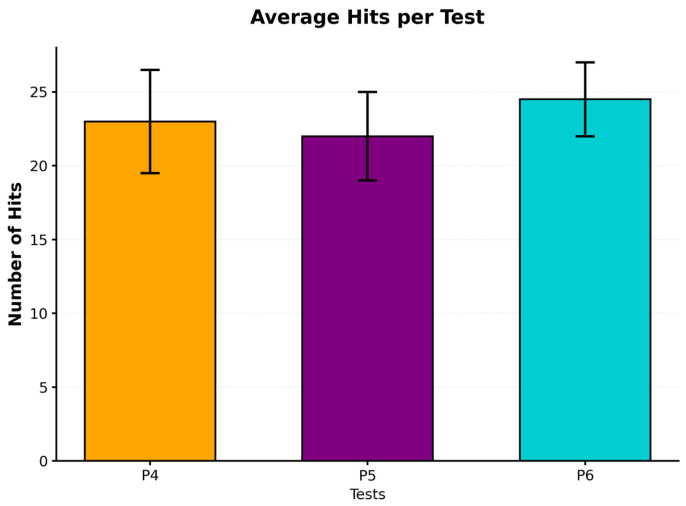
Results of the comparison of average hits per test.

Figure 5 compares average hits per test.

### 3.5. Analysis of Variance (ANOVA)

#### 3.5.1. Time Tests

The one-way ANOVA for the time tests (P1, P2, P3) revealed no significant differences between groups (F = 0.350, *p* = 0.705), indicating that reaction times are consistent across all execution modalities.

#### 3.5.2. Hit Tests

The ANOVA for the hit tests (P4, P5, P6) revealed highly significant differences (F(2, 174) = 9.30, *p* < 0.001, η^2^ = 0.096; η^2^: effect size (partial eta-squared)), indicating that the execution modality has an essential influence on response accuracy.

### 3.6. Visualization of Results

**Figure 6:**
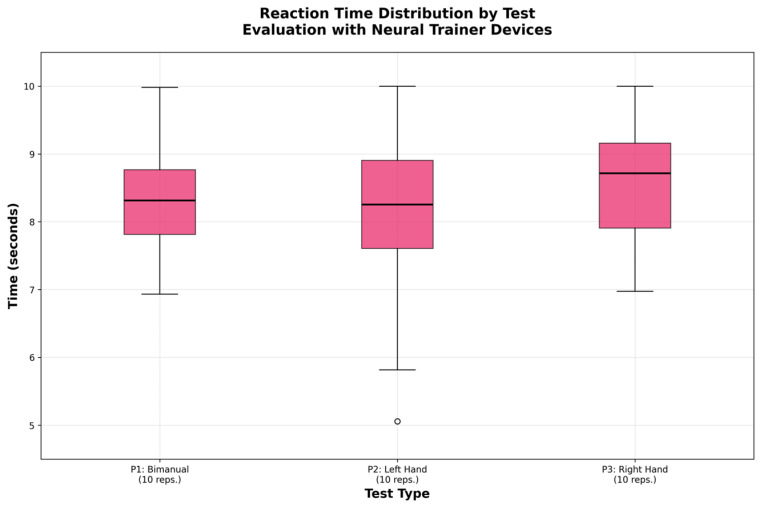
Distribution of reaction times per test.

**Figure 7:**
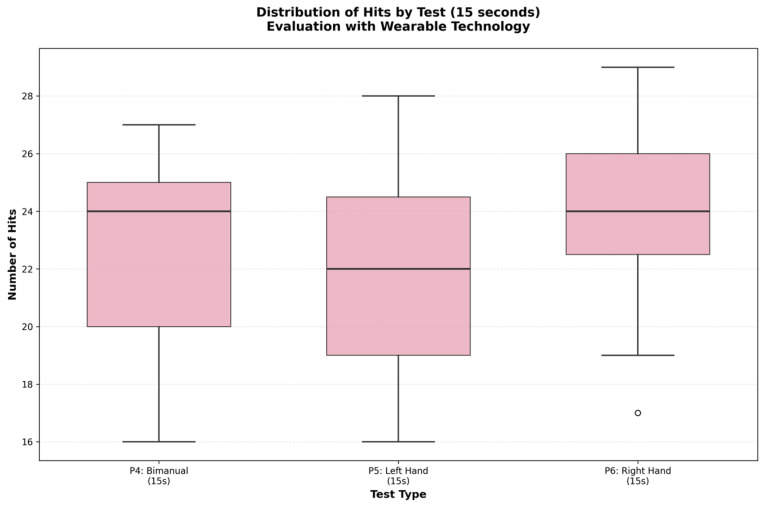
Distribution of reaction hits per test.

Figure 6 and Figure 7 show the distribution of the data using box plots. Figure 6 demonstrates the homogeneity in reaction times across the different modalities, while Figure 7 illustrates the differences in accuracy, particularly the superiority of the right hand.

## 4. Discussion

The results of the present study demonstrate the effectiveness of Neural Trainer devices in objectively assessing reaction time in schoolchildren. The main findings include (1) homogeneity in reaction times across performance modalities, (2) significant superiority of the right hand in accuracy, and (3) moderate correlations between the tests, suggesting standard components in hand–eye coordination.

The absence of significant differences in reaction times across modalities (*p* = 0.705) suggests that the temporal component of the motor response is relatively independent of laterality or bimanual use. This finding is consistent with previous studies, which indicate that simple reaction times are primarily determined by central neural processing factors [16].

The absence of significant differences in reaction times between hands (*p* = 0.716), in contrast to substantial differences in accuracy (*p* < 0.001), reflects the complexity of the underlying neuromotor processes. Children with neurodevelopmental conditions and cerebral palsy commonly display interlimb asymmetries and altered lateralization patterns; instrumented bimanual tasks and neural measures reveal both behavioral and cortical correlates of these asymmetries [17,18].

The moderate correlations observed among the tests suggest consistency in participants’ motor performance across different modalities, which may indirectly reflect integrated visuomotor functioning rather than a direct measure of hand–eye coordination. This supports the usefulness of a comprehensive battery of tests for a thorough assessment [19].

These findings suggest potential applications of the Neural Trainer protocol in educational and clinical contexts for monitoring neuromotor performance; however, further validation in populations with motor or cognitive impairments is required before diagnostic use can be recommended. The use of sensor-based systems such as Neural Trainer may facilitate objective monitoring of neuromotor performance in future longitudinal studies designed to assess developmental trajectories.

Additionally, in [20], a combined motor precision and visual art program was implemented with schoolchildren, resulting in improvements in manual dexterity, motor precision, and reaction time compared with the control group. This suggests that targeted training can differentially improve accuracy and RT, consistent with the dissociation observed in our results.

Neural Trainer devices offer substantial advantages over traditional methods, including millisecond temporal accuracy, automatic data recording, protocol standardization, real-time analysis, and reduced human measurement errors. A scoping review [21] of mobile health technology for assessing and intervening on upper extremity motor function in children with motor impairments highlights that emerging technologies offer unprecedented opportunities for continuous, objective evaluation. Similarly, the study [22] demonstrated that multi-sensor wearable sensors can provide a quantified assessment of gross motor skills in infants, setting a precedent for longitudinal developmental monitoring.

This study has several limitations that should be considered. For example, the cross-sectional design does not allow for establishing causal relationships or evaluating temporal changes; the non-probability sampling selection limits the generalization of results; variables such as socioeconomic status, prior experience, or general motor development were not controlled for; and the lack of longitudinal assessments at follow-up prevent analysis of the temporal stability of the measurements. Although Neural Trainer devices have proven effective for assessment, dependence on specific technology raises questions about standardization and comparability across studies. Tang et al. [23] developed a multimodal fusion network based on wearable sensors for automated assessment of gait dysfunction in children with cerebral palsy, highlighting the importance of methodological standardization to enable cross-study synthesis and clinical translation. A direct analysis of hand–eye interaction would require additional instrumentation such as eye-tracking, which was beyond the scope of this study.

The observed reaction times (8.69–8.90 s for 10 repetitions) are consistent with ranges reported in previous literature for similar populations [24]. The lateral differences in accuracy also coincide with studies documenting the advantages of the dominant hand in coordination tasks [25]. Hand dominance was not included as a control variable; therefore, the observed superiority of the right hand in the hit tests (P5–P6) may reflect the predominance of right-handed participants in the sample rather than a universal lateralization effect.

Future studies should include dominant-hand classification (e.g., using the Edinburgh Handedness Inventory) to confirm whether the performance differences are associated with neuromotor asymmetry or habitual dominance. The results suggest several lines of future research, including longitudinal studies to assess the temporal development of these abilities, analyses of predictive factors of performance, validation of protocols in clinical populations, the development of artificial intelligence algorithms for predictive analysis, and integration with other neuromotor assessment technologies.

The convergence of our results with recent literature reinforces the validity of this technological approach. This convergence aligns with emerging research on neural interface technologies that aim to bridge brain, motor, and sensory systems through wearable and intelligent devices [13], highlighting the potential of such tools for real-time assessment and adaptive neurofeedback in educational and clinical environments. It suggests a promising future for the objective evaluation and monitoring of neuromotor development in schoolchildren. However, successful clinical translation will require continued efforts in methodological standardization, the development of population norms, and validation in diverse contexts.

## 5. Conclusions

The present study demonstrates that Neural Trainer devices are effective and accurate tools for assessing reaction time in schoolchildren. Key findings include the technological feasibility of Neural Trainer devices, which provide objective, precise, and reproducible measurements of reaction time in the school population; the lateral difference showed a significant superiority of the right hand in terms of accuracy (*p* < 0.001), while reaction times were similar across modalities. Interest correlations (r = 0.392–0.533) suggest common factors in hand–eye coordination, supporting the use of comprehensive assessment batteries. Reference values were established for the study population, facilitating future diagnostic and follow-up assessments and clinical applicability of the developed protocols. These protocols can be applied in educational and clinical settings to identify neuromotor difficulties early. These results contribute to the development of evidence-based neuromotor assessment methodologies and provide a foundation for designing targeted interventions in school-aged populations. The implementation of these technologies can significantly enhance diagnostic accuracy and facilitate the monitoring of neuromotor development in children.

## Figures and Tables

**Table 1:** Descriptive statistics for the reaction time tests.

Tests	Description	N	Mean	S.D.	Min	Max	Q1	Q3	IC95_inf	IC95_sup
P1	Time 10 reps, bimanual.	59	8.6949	1.4414	6	10	8	10	8.3271	9.0627
P2	Time 10 reps, left hand.	59	8.8983	1.2958	5	10	8	10	8.5676	9.2289
P3	Time 10 reps, right hand.	59	8.8305	1.2885	6	10	8	10	8.5017	9.1593
P4	Hits, bimanual.	59	22.8983	3.1000	16	27	20	25	22.1072	23.6893
P5	Hits, left hand.	59	22	3.3987	16	28	19	24.5	21.1327	22.8672
P6	Hits, right hand.	59	24.4237	2.7241	17	29	22.5	26	23.7286	25.1188

N = Number of participants, S.D. = Standard Deviation, Min = Minimum Value, Max = Maximum Value, Q1 = First Quartile (25th percentile), Q3 = Third Quartile (75th percentile), IC95_inf = Lower limit of the 95% Confidence Interval for the mean, IC95_sup = upper limit of the 95% Confidence Interval for the mean.

## Data Availability

The data presented in this study are available on request from the corresponding author due to ethical reasons.
